# Clinical Versus Dermoscopic Evaluation of Tumor Margins Prior to Surgical Excision—A Systematic Review

**DOI:** 10.3390/jcm14176014

**Published:** 2025-08-26

**Authors:** Anestis Chrysostomidis, Evangelos Kostares, Antonios Saramantos, Konstantinos Lallas, Aimilios Lallas, Maria Kantzanou, Ioannis Tilaveridis, Athanassios Kyrgidis

**Affiliations:** 1 Department of Oral and Maxillofacial Surgery, Aristotle University of Thessaloniki, 54124 Thessaloniki, Greece; a.chrisostomidis@gmail.com (A.C.); saramantosant@gmail.com (A.S.); jtilaver@yahoo.gr (I.T.); 2 First Department of Dermatology, School of Medicine, Faculty of Health Sciences, Aristotle University, 54124 Thessaloniki, Greece; emlallas@gmail.com; 3 Department of Microbiology, Medical School, National and Kapodistrian University of Athens, 11527 Athens, Greece; kostevang@med.uoa.gr (E.K.); maria.kantzanou@gmail.com (M.K.); 4 Department of Medical Oncology, School of Medicine, Faculty of Health Sciences, Aristotle University, 54124 Thessaloniki, Greece; koplallas@gmail.com

**Keywords:** dermoscopy, surgical margins, basal cell carcinoma, squamous cell carcinoma, non-melanoma skin cancers, systematic review

## Abstract

**Background/Objectives**: Accurate surgical margin delineation is essential in the treatment of non-melanoma skin cancers (NMSCs), particularly basal cell carcinoma (BCC) and cutaneous squamous cell carcinoma (cSCC), to reduce recurrence and metastasis. Dermoscopy improves diagnostic accuracy for skin tumors, but its utility for preoperative margin assessment remains underexplored. To compare dermoscopy-guided versus clinical visual inspection for preoperative margin assessment in NMSC excision, focusing on histological clearance rates and surgical outcomes. **Methods**: This systematic review and meta-analysis followed PRISMA 2020 guidelines. MEDLINE, Cochrane CENTRAL, Scopus, and Web of Science were searched from inception to 1 July 2025. Eligible studies included adult patients undergoing surgical excision of histologically confirmed BCC or cSCC, with preoperative margin evaluation using either dermoscopy or clinical examination. The primary outcome was the rate of complete histological excision. Study quality was assessed using the Newcastle–Ottawa Scale. A random-effects meta-analysis using the Freeman–Tukey transformation was performed. **Results**: Nine cohort studies comprising 900 NMSC lesions were included. Dermoscopy-guided excision demonstrated pooled histological clearance of 98.7% (95% CI: 97–99.8%), compared to 80–94% with clinical assessment. Moderate heterogeneity was observed (I^2^ = 42%). However, variability in study design and limited data for cSCC restricted broader conclusions. **Conclusions**: Dermoscopy may enhance margin assessment and histological clearance in NMSC surgery, especially for BCC. Further standardized, high-quality studies are needed to confirm its role in surgical planning and extend evidence to SCC.

## 1. Introduction

Non-melanoma skin cancers (NMSCs)—including basal cell carcinoma (BCC) and cutaneous squamous cell carcinoma (cSCC)—are the most common malignancies worldwide. NMSC represents the most common human cancer, and the incidence is rising worldwide. The estimated incidence of NMSC in the USA and Australia is more than 1,000,000 and 480,000 cases per year, respectively [1,2]. Incomplete excision of non-melanoma skin cancers (NMSCs) occurs in approximately 10% of cases, with higher rates observed in anatomically complex or cosmetically sensitive regions. Such incomplete resections are clinically significant, as they are associated with increased risk of local recurrence, the need for additional surgical interventions, and elevated healthcare utilization. These considerations highlight the critical importance of precise preoperative margin delineation to optimize oncologic outcomes and reduce the likelihood of repeat procedures [3,4,5].

Dermoscopy involves the use of magnification and polarized light to enhance visualization of morphological features and peripheral borders of suspicious cutaneous lesions. It is endorsed by the National Institute for Health and Care Excellence (NICE) as an adjunct to clinical assessment for the diagnosis of skin malignancies [6,7,8,9,10]. However, no formal guidelines currently address its role in surgical planning or margin delineation. By revealing subclinical features—such as pigment networks, vascular patterns, or architectural asymmetry—dermoscopy may facilitate more accurate identification of tumor boundaries and inform adjustments to excision margins that would otherwise be missed by visual inspection alone. Meta-analyses demonstrate that dermoscopy improves sensitivity from 79% to 93% and specificity from 77% to 99% in detecting BCC [11,12]. Despite extensive use in diagnosis, its potential for intraoperative margin mapping and guiding surgery is less established. Standard clinical margin determination, while widely practiced, has significant limitations: incomplete excision rates of up to 17% have been reported in sensitive anatomical regions, such as the periorbital area [13].

Several studies have shown that dermoscopy more accurately delineates tumor borders than clinical assessment alone. A prospective study of 88 BCCs found dermoscopic margins differed from clinical margins in 67% of cases and regularly revealed subclinical tumor extensions—such as pink-white areas and short telangiectasias—extending beyond clinically defined borders [11]. Similarly, cSCC margin delineation showed 47.8% discordance between dermoscopic and clinical borders, and presurgical dermoscopy-guided resection using an 8 mm margin achieved 98–100% histological clearance even when visible residuals were uncertain [14].

A systematic review and meta-analysis concluded that using dermoscopy to guide margin marking significantly reduces incomplete excision rates in NMSC surgical management [1]. Moreover, dermoscopic margin assessment has been incorporated into Mohs micrographic surgery protocols, with “DerMohscopy” demonstrating reduced positive lateral margins, fewer stages, and smaller defects [15]. Mohs micrographic surgery (MMS) is widely recognized as the most effective technique for achieving complete excision of skin tumors, with excellent cure rates for both initial and recurrent cases [16,17]. Nevertheless, its use is limited by practical constraints, including the need for extended operative time and specialized surgical training. Consequently, standard surgical excision using predetermined margins continues to be an appropriate treatment option for well-circumscribed basal cell carcinomas (BCCs) with low-risk histological subtypes. What is more, its benefit during presurgical mapping is not without controversy. A recent randomized study comparing dermoscopy versus clinical assessment in Mohs surgery found no significant difference in the number of stages or initial margin accuracy between groups [18]. This suggests variable results, perhaps due to differences in operator experience, tumor subtype, or dermoscopic criteria used.

Consequently, this review aims to critically synthesize the evidence comparing dermoscopic vs. clinical visual evaluation, focusing on (1) accuracy of border detection, (2) histological margin clearance, and (3) dermoscopy’s role in pre-operative surgical planning and oncological radicality. This study is aimed to explore whether dermoscopy-guided assessment is associated with higher rates of histologically clear margins compared to clinical inspection alone by systematically reviewing currently available literature and to provide the first quantitative synthesis of relevant studies.

## 2. Materials and Methods

### 2.1. Protocol and Registration

This systematic review and meta-analysis was conducted in accordance with the Preferred Reporting Items for Systematic Reviews and Meta-Analyses (PRISMA) guidelines [19]. The PRISMA checklist is available in the Supplementary Materials (Supplementary Table S1).

### 2.2. Eligibility Criteria

Studies were included if they met the following criteria:

- Study design: Interventional studies (randomized and non-randomized controlled trials) and observational studies (such as cohort studies, cross-sectional studies, and case–control studies).
- Population: Adult patients (≥18 years) undergoing surgical excision of histologically confirmed non-melanoma skin cancers (BBC and/or SCC). Intervention: Preoperative margin evaluation using dermoscopy.
- Comparator: Preoperative margin evaluation using clinical visual inspection alone (naked-eye examination).
- Outcomes: Primary outcome was the rate of complete excision (histologically clear margins).
- Language: Only studies published in English were included.
- Publication type: Peer-reviewed articles. Abstracts, case reports, case-series < 5 patients editorials, and reviews were excluded.

### 2.3. Information Sources and Search Strategy

A comprehensive literature search was performed in the following electronic databases: MEDLINE (via PubMed), Cochrane CENTRAL, Web of Science, and Scopus, from inception to 1 July 2025. The search strategy combined terms and synonyms for “non-melanoma skin cancer”, “dermoscopy”, “clinical examination”, and “surgical excision”. Reference lists of relevant studies and reviews were also screened. A full search strategy is provided in Supplementary Material (Supplementary Table S2).

### 2.4. Study Selection

Two independent reviewers screened all titles and abstracts for eligibility. Full texts of potentially relevant studies were then assessed in duplicate. Disagreements were resolved by consensus or by a third reviewer. The study selection process is detailed in the PRISMA flow diagram.

### 2.5. Data Extraction

Data were extracted independently by two reviewers using a standardized data extraction form. The following information was collected:

- Study characteristics: author, year, country, study design;
- Patient demographics and tumor characteristics;
- Method of margin evaluation (dermoscopy vs. clinical);
- Surgical technique (e.g., standard excision);
- Primary outcomes as defined above;
- Where data were missing or unclear, study authors were contacted for clarification.

### 2.6. Risk of Bias Assessment

Study quality was independently evaluated by two researchers using the Newcastle–Ottawa Scale (NOS), developed by the Universities of Newcastle and Ottawa. This tool assesses selection, comparability, and outcome/exposure. Scores of 7–9 indicated high quality (low risk of bias), 4–6 moderate quality, and 0–3 low quality (high risk of bias).

### 2.7. Data Synthesis and Statistical Analysis

Model Use: Statistical analyses were performed using RStudio (version 2022.12.0 + 353). The meta-analysis was conducted with the metafor package, applying the Freeman–Tukey double arcsine transformation method.

Heterogeneity and Analyses: Heterogeneity among studies was assessed using Cochran’s Q statistic and its *p*-value, along with visual inspection of the forest plot. The Higgins’ I^2^ statistic and its 95% confidence interval were calculated to estimate the degree of true heterogeneity. I^2^ values of 0–40%, 30–60%, 50–90%, and 75–100% were interpreted as indicating low, moderate, substantial, and considerable heterogeneity, respectively.

## 3. Results

### 3.1. Study Characteristics

**Figure 1:**
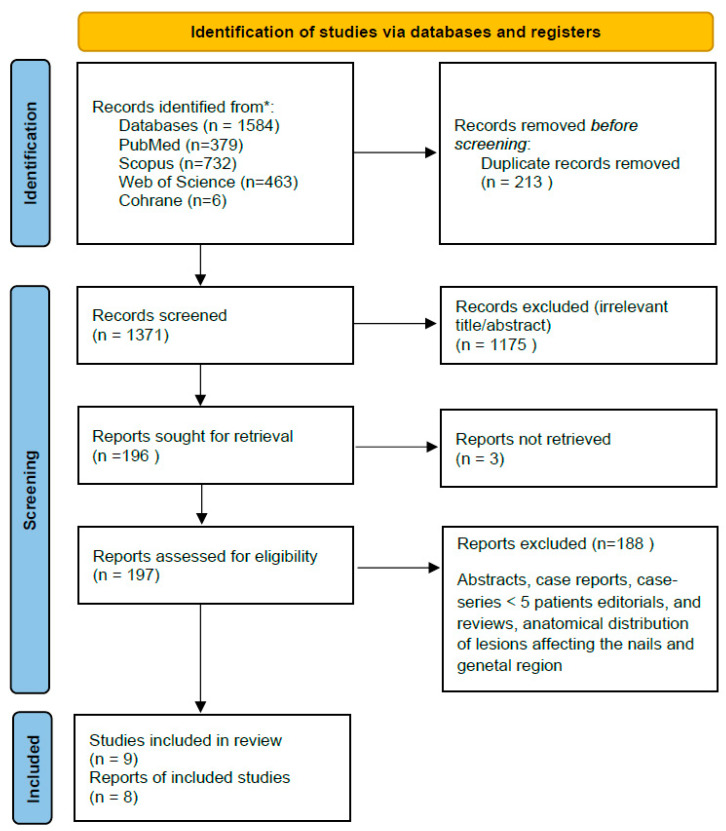
PRISMA flowchart. * MEDLINE (via PubMed), Cochrane CENTRAL, Web of Science, and Scopus, from inception to 1 July 2025.

A total of nine studies were included in this systematic review, comprising data from Europe and Asia [4,11,14,20,21,22,23,24,25]. Figure 1 provides the PRISMA flowchart. The studies were published between 2010 and 2023 and included only cohort designs, either prospective or retrospective. Five studies were conducted in Europe (Italy and Romania) [11,20,21,22,24], and four in Asia (China, Japan, and India) [4,14,23,25]. Sample Sizes ranged from 17 to 288 lesions, with a total of 900 lesions across all studies. BCC was studied in seven studies [4,11,21,22,23,24,25], while SCC was examined in two [14,20]. Mean Age varied between 60.8 and 81 years, with most studies reporting a male predominance (ranging from 33.3% to 64.7%). All of the studies were assessed to be of moderate quality based on the quality assessment [4,11,14,20,21,22,23,24,25]. Table 1 summarizes their descriptive characteristics.

### 3.2. Margin Clearance Rates

Margin clearance rates for BCC ranged from 93.3% (4 mm margin) to 100% (8 mm margin) in one study, while others reported rates of 98.1%, 99.3%, and 100%. One study reported a 94% clearance rate for SCC, while another did not provide specific data.

Two studies compared their results with standard clinical assessments, reporting clearance rates of 93% and 94%, versus 80% and 83%, respectively.

To provide further detail, a comparison across studies reveals consistent findings in favor of dermoscopy for preoperative margin evaluation. In the study by Ito et al. [23], dermoscopy achieved a 99.3% clearance rate across 288 lesions, underscoring its reliability in large sample applications. Similarly, Chen et al. (2022) [4] noted a 98.1% margin clearance rate and emphasized that 16.8% of cases had underestimated margins based on clinical inspection alone—highlighting dermoscopy’s role in detecting subclinical spread.

Carducci et al. (2011, 2013) [20,21], conducted dual studies focusing on both BCC and SCC. In BCC, dermoscopy improved margin clearance from 80% (clinical) to 93%, and in SCC from 83% to 94%, emphasizing dermoscopy’s utility beyond just BCC. Notably, Liu et al. (2023) [14], demonstrated perfect histological clearance in SCC using dermoscopy-guided resection, affirming its value in more aggressive tumors.

The study by Conforti et al. (2020) [11] found that clinical and dermoscopic borders differed in 67% of cases, with dermoscopy frequently identifying deeper extensions. Lupu et al. (2021) [24], confirmed this discordance, despite technical losses in tissue processing.

Smaller studies (Sushil et al., Caresana et al.) [22] corroborated these trends. Even in studies with fewer than 20 cases, dermoscopy consistently yielded clearance rates of 98–100%. Taken together, these data support the hypothesis that dermoscopy enhances surgical planning by better defining tumor peripheries.

Importantly, no studies reported a higher margin clearance rate with clinical inspection over dermoscopy. Recurrence rates were also negligible or zero in dermoscopy-guided groups, suggesting that improved initial clearance may lead to better long-term outcomes.

**Figure 2:**
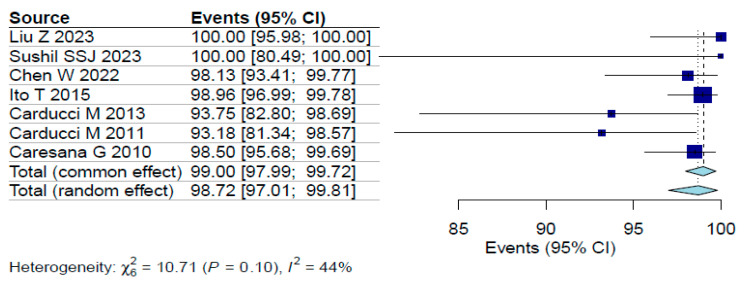
Forest plot [4,7,8,10,11,12,13].

A random-effects meta-analysis was conducted to estimate the pooled proportion of lesions with histologically clear surgical margins following dermoscopy-guided excision. The analysis yielded a pooled proportion of 98.7% (95% CI: 97–99.8%). Moderate heterogeneity was observed across studies, as indicated by an I^2^ value of 42% (*p* < 0.001), as presented in Figure 2. This relatively low heterogeneity strengthens confidence in the findings, suggesting the positive impact of dermoscopy is not overly dependent on study-specific factors. However, caution is still warranted given the lack of randomized trials and standardized methodologies.

### 3.3. Risk of Bias

A moderate risk of bias was identified across the majority of included studies as illustrated in Figure 1. The inherent nature of the intervention, specifically, the application of dermoscopy precluded the possibility of participant or assessor blinding, thereby introducing potential performance and detection bias. Two studies by Carducci et al. [11,12] were designed as non-randomized controlled trials comparing dermoscopy-guided surgical margin delineation with standard naked-eye assessment. The remaining studies employed cross-sectional methodologies, in which dermoscopy was uniformly applied and histological margin clearance was assessed retrospectively. The lack of randomization, blinding, and standardized outcome assessment across these studies compromises the internal validity and limits the generalizability of their findings.

## 4. Discussion

Dermoscopy-assisted margin delineation appears to improve surgical clearance rates for NMSC compared to clinical evaluation alone, particularly in controlled settings it allows visualization of the superficial layers of the skin and provides additional details derived from a microscopic level, which include the architecture, the vascular structures and the distribution of color across the lesion. A detailed comparison of key parameters used in clinical and dermoscopic margin assessment is provided in Table 2 [9,26,27,28,29]. The Cochrane review on the utility of desmoscopy, also other reviews, have commented on the importance of dermoscopy in the diagnosis of nonmelanoma skin neoplasms [30,31]. Non-melanoma skin cancers (NMSCs) predominantly occur on sun-exposed areas, particularly the head and neck, due to cumulative ultraviolet (UV) radiation exposure [13]. Incomplete tumor removal not only increases the risk of local recurrence but, in the case of cutaneous squamous cell carcinoma (cSCC), may also elevate the potential for metastasis. Consequently, additional therapeutic interventions are often necessary to achieve complete disease control. Our findings suggest a strong correlation between the use of dermoscopy and improved surgical outcomes, particularly in terms of achieving histologically clear margins. However, heterogeneity in study design, reporting quality, cancer type, and surgical margins limited direct comparability across studies. The apparent superiority of dermoscopy may be attributed to its ability to visualize tumor margins with higher accuracy, especially when compared to clinical judgment alone, which can often underestimate lateral extensions [15]. Furthermore, dermoscopy offers an immediate, cost-effective, and non-invasive method for real-time margin assessment without delaying surgical workflow [2,27,32]. Understanding how dermoscopy reduces reoperations or recurrence would help justify investments in training and equipment, especially in lower-resource settings. It could bridge disparities in dermatologic surgical care if implemented thoughtfully. Its integration into daily clinical practice does not require significant infrastructure, and its use can be especially helpful in cases where the preservation of aesthetic or functional tissue is critical—such as on the face or periorbital areas.

This systematic review evaluated the comparative effectiveness of dermoscopy versus clinical visual inspection for preoperative margin delineation in non-melanoma skin cancer (NMSC) surgery. The findings suggest that dermoscopy-assisted margin assessment may contribute to higher histological clearance rates compared to clinical evaluation alone, particularly for BCC. However, the evidence remains heterogeneous and limited by methodological variability across studies.

The reviewed studies consistently reported high margin clearance rates for BCC (ranging from 93% to 100%) when dermoscopy was employed. Notably, two cohort studies of Carducci et al. 2011 and 2013 [20,21] demonstrated superior clearance rates with dermoscopy (93% and 94%) compared to standard clinical assessment (40% and 46%). This consistent under-detection by clinical inspection highlights dermoscopy not just as a helpful adjunct, but as a potential new standard in preoperative evaluation. The data support the idea that reliance on clinical judgment alone may now be ethically and medically insufficient in many cases. For squamous cell carcinoma (SCC), the available data were more limited, though one study reported excellent clearance rates of 98–100% when using dermoscopy-guided 8 mm margins. The paucity of SCC-specific data is a significant limitation. Given SCC’s more aggressive nature, future research should address whether dermoscopy’s benefits in BCC extend similarly to SCC, potentially improving oncological safety in a higher-risk group.

These findings align with previous meta-analyses suggesting that dermoscopy reduces incomplete excision rates by identifying subtle tumor extensions—such as pink-white areas and short telangiectasias—that are frequently missed by clinical inspection alone.

The significant discordance observed between dermoscopic and clinical margin assessments provides compelling evidence for dermoscopy’s ability to detect subclinical tumor spread beyond visually apparent borders. This likely explains the higher clearance rates achieved with dermoscopy-assisted excisions. However, it is important to note that one study found no significant difference in Mohs surgery outcomes between dermoscopy and clinical assessment groups, suggesting that factors such as operator expertise and tumor characteristics may moderate dermoscopy’s benefits.

However, broader adoption of this technique will require additional validation and standardization. The development of consensus criteria for dermoscopic margin assessment (focusing on features like vascular patterns and ulceration) and implementation of structured training programs could help improve consistency across different clinical settings [3,33].

Another potential application lies in surgical education. As dermoscopy requires pattern recognition and interpretive skill, incorporating its use into dermatologic and surgical training could yield broader benefits for tumor detection and management [34]. Furthermore, dermoscopic mapping can be photo-documented and reviewed, making it a useful tool in quality assurance and surgical planning audits.

### Limitation

Several important limitations must be acknowledged when interpreting these findings. Most studies are observational in nature, lacking randomization or blinded outcome assessment. The moderate heterogeneity among included studies—encompassing variations in study design, surgical techniques, excision margins and outcome definitions make cross-study comparisons challenging. Due to inconsistent data on standard deviation, effect sizes, or event rates in control groups, a quantitative meta-analysis could not be conducted for all outcomes. The generalizability of findings may also be affected by differences in lesion location, histologic subtype, and operator proficiency. Methodological quality was inconsistent, with most observational studies lacking robust control groups or detailed randomization procedures. Furthermore, the validity of our findings was limited by the quality of the included studies, as several were assessed as being of low or unclear methodological quality. The evidence base for SCC management was particularly sparse, with only two cohort studies conducted on this entity thus far [14,20], representing a significant knowledge gap given this tumor type’s greater metastatic potential, warranting further investigation. Additionally, the performance of dermoscopy is likely highly dependent on operator experience, a factor that was rarely documented in the reviewed studies. Moreover, this systematic review has not been registered in PROSPERO, which may be a potential source of bias.

To advance the field, future research should prioritize multicenter randomized controlled trials, especially those comparing dermoscopy to other imaging modalities such as reflectance confocal microscopy or high-resolution ultrasound [17,24,26,35,36]. Cost–benefit analyses would also help determine the feasibility of widespread dermoscopy adoption, particularly in low-resource settings. Such future research should focus on addressing these limitations through well-designed prospective RCTs that compare dermoscopy-guided versus clinical margin assessment using standardized excision criteria and blinded histopathological evaluation. Investigations into the cost-effectiveness of dermoscopy would be valuable, particularly examining whether its use reduces the need for re-excisions or decreases recurrence rates enough to justify the required training investment. There is also a pressing need for SCC-specific studies to better understand dermoscopy’s role in managing these higher-risk tumors. What is more, while dermoscopy appears to enhance preoperative margin assessment, its optimal use requires standardized protocols, validated training modules, and broader clinical integration. The accumulating evidence, however, clearly supports dermoscopy’s role in improving both oncologic control and cosmetic outcomes in NMSC excision. The included studies in this review demonstrate generally high margin clearance rates for non-melanoma skin cancers, particularly BCC. This review provides a foundation for guideline committees to reconsider existing protocols. The evidence supports dermoscopy’s inclusion as a recommended, possibly even standard, tool for preoperative planning in NMSC surgery. However, heterogeneity in study designs, reporting standards, and missing methodological details suggest the need for more standardized research in this area.

## 5. Conclusions

In conclusion, the current evidence suggests that the integration of dermoscopy as an adjunctive tool for surgical margin delineation offers a rapid and reliable method for enhancing intraoperative decision-making. Its ease of implementation within surgical settings makes it a practical addition to standard practice and may contribute to improved patient outcomes by decreasing the likelihood of histologically involved margins in non-melanoma skin cancer (NMSC) excisions. While these findings support the adjunctive use of dermoscopy in preoperative planning, especially for high-risk tumors, its universal implementation will require additional high-quality research to establish standardized protocols and clarify its benefits across different clinical contexts. Clinicians should consider incorporating dermoscopy into their practice while remaining mindful of its current limitations and the need for ongoing skill development.

The key takeaway from this review is that dermoscopy represents a promising advance in NMSC management, but its full potential will only be realized through continued research efforts aimed at optimizing and standardizing its application in surgical practice.

## Figures and Tables

**Table 1:** Detailed characteristics of the studies that were included in the evaluation.

First Author	Year of Publication	Country	Study Design	Study Period	Sample Size	Lesions	Mean Age (Years)	Males (%)	Cancer Type (BCC, SCC)	Comparison Group (Standard Visual, etc.)	Margin Clearence	Margin Clearance Rate (%)	Recurrences	Risk of Bias Assessment	Comments/Notes
Liu Z [4]	2023	China	Cohort	2016–2022	90	90	74	56.7	SCC	NA	90	100%	0%	Moderate	Wider dermoscopic borders compared to those identified visually (38.9%)
Sushil SSJ [7]	2023	India	Cohort	2015–2020	17	17	60.8	64.7	BCC	NA	17	100%	0%	Moderate	Preoperative dermoscopy was useful in correlating the clinical subtypes with the final histopathological diagnosis in all the cases
Chen W [8]	2022	China	Cohort	2016–2020	107	107	64.2	61.7	BCC	NA	105	98.1	0	Moderate	Eighten of 107 (16.8%) patients showed that the visual margin was inadequate compared to the dermoscopy-detected margin
Lupu M [9]	2021	Romania	Cohort	2018–2020	18	20	71.5	33.3	BCC	NA	21	72.41	NA	Moderate	32 margins in 20 BCC were explored, 3 margins destroyed during tissue processing, leaving 29 margins in the final analysis
Conforti C [3]	2020	Italy	Cohort	2018–2019	88	88	72.8	57.9	BCC	NA	NA	NA	NA	Moderate	Differences between Clinical and Dermoscopic Margins
Ito T [10]	2015	Japan	Cohort	2006–2013	263	288	71.2	46	BCC	NA	285	99.3	0%	Moderate	Dermoscopically determined borders almost exactly corresponded to the histopathological findings
Carducci M [11]	2013	Italy	Cohort	2008–2011	48	48	81	54.1	SCC	46 (clinical)	45	94%	0%	Moderate	Margin possitivity rate in clinical detection group was significantly higher (17%) than in dermoscopic group (6%)
Carducci M [12]	2011	Italy	Cohort	2008–2009	44	44	71.8	52.2	BCC	40 (clinical)	41	93%	NA	Moderate	Margin possitivity rate in clinical detection group was significantly higher (20%) than in dermoscopic group (7%)
Caresana G [13]	2010	Italy	Cohort	2007–2009	200	200	NA	NA	BCC	NA	197	98.50%	0.00%	Moderate	In 69 cases (34.5%) dermoscopic evaluation showed a larger peripheral extension, compared to clinical measurements
Liu Z [4]	2023	China	Cohort	2016–2022	90	90	74	56.7	SCC	NA	90	100%	0%	Moderate	Wider dermoscopic borders compared to those identified visually (38.9%)
Sushil SSJ [7]	2023	India	Cohort	2015–2020	17	17	60.8	64.7	BCC	NA	17	100%	0%	Moderate	Preoperative dermoscopy was useful in correlating the clinical subtypes with the final histopathological diagnosis in all the cases
Chen W [8]	2022	China	Cohort	2016–2020	107	107	64.2	61.7	BCC	NA	105	98.1	0	Moderate	Eighten of 107 (16.8%) patients showed that the visual margin was inadequate compared to the dermoscopy-detected margin
Lupu M [9]	2021	Romania	Cohort	2018–2020	18	20	71.5	33.3	BCC	NA	21	72.41	NA	Moderate	32 margins in 20 BCC were explored, 3 margins destroyed during tissue processing, leaving 29 margins in the final analysis
Conforti C [3]	2020	Italy	Cohort	2018–2019	88	88	72.8	57.9	BCC	NA	NA	NA	NA	Moderate	Differences between Clinical and Dermoscopic Margins
Ito T [10]	2015	Japan	Cohort	2006–2013	263	288	71.2	46	BCC	NA	285	99.3	0%	Moderate	Dermoscopically determined borders almost exactly corresponded to the histopathological findings
Carducci M [11]	2013	Italy	Cohort	2008–2011	48	48	81	54.1	SCC	46 (clinical)	45	94%	0%	Moderate	Margin possitivity rate in clinical detection group was significantly higher (17%) than in dermoscopic group (6%)
Carducci M [12]	2011	Italy	Cohort	2008–2009	44	44	71.8	52.2	BCC	40 (clinical)	41	93%	NA	Moderate	Margin possitivity rate in clinical detection group was significantly higher (20%) than in dermoscopic group (7%)
Caresana G [13]	2010	Italy	Cohort	2007–2009	200	200	NA	NA	BCC	NA	197	98.50%	0.00%	Moderate	In 69 cases (34.5%) dermoscopic evaluation showed a larger peripheral extension, compared to clinical measurements

**Table 2:** Key differences between clinical (visual) and dermoscopy-guided preoperative margin assessment in non-melanoma skin cancer.

Parameter	Clinical (Visual/Naked-Eye) Inspection	Dermoscopy-Guided Assessment
Tool used	Unassisted visual examination	Handheld dermoscope (polarized or non-polarized light)
Magnification	None or minimal (unaided eye)	Typically 10× magnification
Light source	Ambient/room light	Polarized or cross-polarized light
Border definition	Based on visible color, texture, elevation	Enhanced visualization of subclinical tumor margins
Common features evaluated	Lesion size, color, induration, ulceration, surface irregularity	Arborizing vessels, blue-gray ovoid nests, leaf-like areas, pink-white areas, short telangiectasias, pigment network, peripheral structures
Margin determination	Estimation based on lesion appearance and palpation	Identification of subtle extensions beyond visible border
Operator dependency	Subjective, based on clinical experience	Subjective but aided by pattern recognition and dermoscopic criteria
Documentation	Often non-standardized	Can be photo-documented and reproducible
Limitations	May miss subclinical extensions; prone to underestimation in cosmetically sensitive sites	Requires training; interobserver variability in interpretation
Clinical utility	Standard method; quick and widely used	Adjunctive method; enhances margin precision especially in BCC

## Data Availability

No new data were created or analyzed in this study.

## Supplementary Materials

The following supporting information can be downloaded at: https://www.mdpi.com/article/10.3390/jcm14176014/s1, Table S1. PRISMA Checklist; Table S2. Literature search.
