# Supplementary material for: Clinical Versus Dermoscopic Evaluation of Tumor Margins Prior to Surgical Excision—A Systematic Review
Source: J Clin Med. 2025 Aug 26;14(17):6014. doi: 10.3390/jcm14176014 (PMC12429733; doi:10.3390/jcm14176014)
Supplement: Supplementary file 1 [file jcm-14-06014-s001.zip › Supplementary Table S2.pdf]

Supplementary Table 2. Literature search

Database	Search Terms	Filters
Medline (Pubmed)	("preoperat*"[All Fields] OR "oper*"[All Fields]) AND "dermoscop*"[All Fields]	-
Scopus	(preoperat* OR oper*) AND dermoscop*	Article title, Abstract, Keywords
Web of Science	(preoperat* OR oper*) AND dermoscop*	-
Cochrane Central	(preoperat* OR oper*) AND dermoscop*	-
